# An unusual and challenging case of HIV-associated primary CNS Lymphoma with Hodgkin-like morphology and HIV encephalitis

**DOI:** 10.1186/s13000-015-0387-9

**Published:** 2015-09-02

**Authors:** Isaac E. Lloyd, Parker W. Clement, Karen L. Salzman, Randy L. Jensen, Mohamed E. Salama, Cheryl A. Palmer

**Affiliations:** Department of Pathology, University of Utah, 15 North Medical Drive East, Suite #1100, Salt Lake City, UT 84112 USA; Department of Radiology, University of Utah, 30 N 1900 E, Salt Lake City, UT 84132 USA; Department of Neurosurgery, University of Utah, 175 N. Medical Drive East, Salt Lake City, UT 84132 USA; Department of Pathology, University of Utah and ARUP Laboratories, ARUP Laboratories, 500 Chipeta Way, Salt Lake City, UT 84108-1221 USA; Department of Pathology, University of Utah, Huntsman Cancer Institute, 1950 Circle of Hope Drive, N3150, Salt Lake City, UT 84112 USA

**Keywords:** Primary Central Nervous System Lymphoma (PCNSL), Human Immunodeficiency Virus (HIV), Encephalitis

## Abstract

HIV-associated primary CNS lymphomas are well-recognized, almost exclusively EBV-driven neoplasms with poor clinical prognosis. We report a challenging, atypical case of an HIV-associated lymphoproliferative disorder with unusual morphologic features reminiscent of Hodgkin Lymphoma, accompanied by HIV encephalitis. A 52-year-old male presented with acute seizures after seven months of progressive neurocognitive decline that was clinically diagnosed as progressive supranuclear palsy. Clinical work-up revealed HIV infection along with two ring-enhancing lesions in the brain on MRI, and negative CSF EBV testing. Subsequent biopsy showed well-demarcated hypercellular regions in the brain comprised of scattered Reed-Sternberg-like cells in a background of small to medium-sized lymphocytes exhibiting focal angiocentricity and geographic necrosis. The atypical cells were positive for CD20, EBV, and CD79a, and negative for CD45, GFAP, CD15, CD30, and p24. These cells were admixed with numerous CD68-positive cells. The adjacent brain showed classic features of HIV encephalitis with perivascular, CD68 and p24-positive multinucleated giant cells. This case illustrates several diagnostic pitfalls in the work-up of HIV-associated brain lesions, as well as reporting a unique histomorphology for an HIV-related primary CNS lymphoproliferative disorder.

## Background

Primary CNS lymphoma (PCNSL) is fairly common in HIV-infected patients, affecting as many as 10 % of Acquired Immunodeficiency Syndrome (AIDS) patients in some autopsy studies [1]. It often presents late in the disease process in the setting of severe immunosuppression, and is almost always associated with coincident Epstein-Barr virus (EBV) infection [2, 3]. PCNSL typically presents as a deep, intracranial mass lesion, sometimes ring-enhancing on imaging, and is often difficult to differentiate from toxoplasmosis radiologically. Histologic diagnosis is traditionally sought only in patients who demonstrate lesional progression following a trial of anti-toxoplasmosis therapy [2]. In recent years, EBV DNA testing in CSF has also been promoted as a useful tool for the diagnosis of PCNSL [4]. Histologically, the diagnosis is based on identification of infiltrating, dyscohesive, atypical lymphocytes, often arranged in angiocentric aggregates, and possibly exhibiting areas of necrosis. If associated with HIV infection, multinucleated giant cells, largely in perivascular locations, may be seen throughout the brain and are diagnostic of HIV encephalitis. Immunophenotyping of the atypical cells by immunohistochemistry or flow cytometry is crucial for appropriate diagnosis and classification. Final pathologic classification of these tumors follows the World Health Organization classification scheme for hematopoietic neoplasms [5], the majority being classified as AIDS-related diffuse large B-cell lymphomas (DLBCL) [6, 7]. We present the following unique case of an HIV-associated PCNSL with striking Hodgkin lymphoma-like morphology, along with its diagnostic challenges.

## Case presentation

**Fig. 1:**
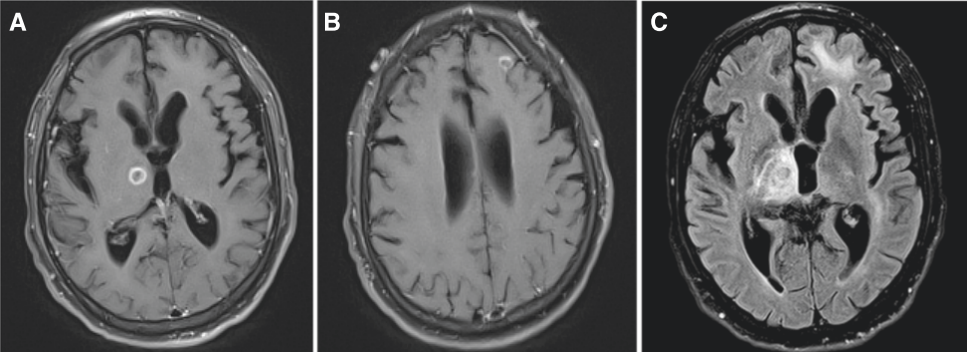
Magnetic Resonance Images. Axial T1 post-contrast Magnetic Resonance Images (MRI) of the patient’s ring-enhancing lesions in the right thalamus (**a**) and left frontal lobe (**b**) with central low signal suggesting necrosis. Axial FLAIR image at the level of the thalamic lesion (**c**) shows edema surrounding the lesion in the right thalamus and left frontal lobe as hyperintense signal. There is mild diffuse cerebral volume loss which is a common finding in HIV encephalitis

A 52-year-old male with no significant past medical history was admitted for seizures after 7 months of cognitive and functional decline, 50-kilogram weight loss, episodic seizures, paranoia, and delusions. A contrast MRI revealed two ring-enhancing lesions in his brain, in the left frontal lobe and right thalamus (Fig. 1).

The patient underwent an extensive clinical work-up including an unremarkable lumbar puncture and negative testing for cryptococcus antigen, EBV (MGB Alert EBV Probe, MGB Alert Primers, Elitetech/Epoch Biosciences, Princeton, NJ, USA; Limit of Detection in CSF: 500 copies/mL), cytomegalovirus (CMV), herpes simplex virus (HSV), varicella zoster virus (VZV), toxoplasmosis, cysticercosis, oligoclonal bands and myelin basic protein, bacterial and fungal cultures, and flow cytometry for lymphoma. However, he tested positive for HIV by quantitative PCR (COBAS Ampliprep/COBAS TaqMan HIV-1 Test v2.0, Roche Diagnostics, Indianapolis, IN, USA) and combination antigen/antibody assay with confirmatory western blot (GS HIV Combo Ag/Ab EIA, GS HIV-1 Western Blot, Bio-Rad Clinical Diagnostics, Hercules, CA, USA), and had a CD4 T-cell count of 22 cells/μL. CT scan of the chest showed diffuse nodular opacities, and a bronchoalveolar lavage was positive for *Pneumocystis jiroveci*. No other significant abnormalities were seen on whole-body imaging. The patient was diagnosed with Acquired Immunodeficiency Syndrome (AIDS) and empirically treated for toxoplasmosis infection of the brain. Repeat MRI of the brain a week later revealed no improvement in the brain lesions, and stereotactic-guided biopsy was performed of the left frontal lesion.

The biopsy was sent for intraoperative consultation via frozen section, and showed mostly mildly astrogliotic brain parenchyma with a small focus of hypercellularity. This focus was comprised of a mixture of small and large epithelioid cells with nuclear pleomorphism, suspicious for a neoplastic process. There was no evidence of acute inflammation or microorganisms such as toxoplasmosis. A portion of the specimen was submitted for flow cytometric evaluation and was found to have no evidence of monoclonality or non-Hodgkin lymphoma, but may not have contained lesional tissue.

**Fig. 2:**
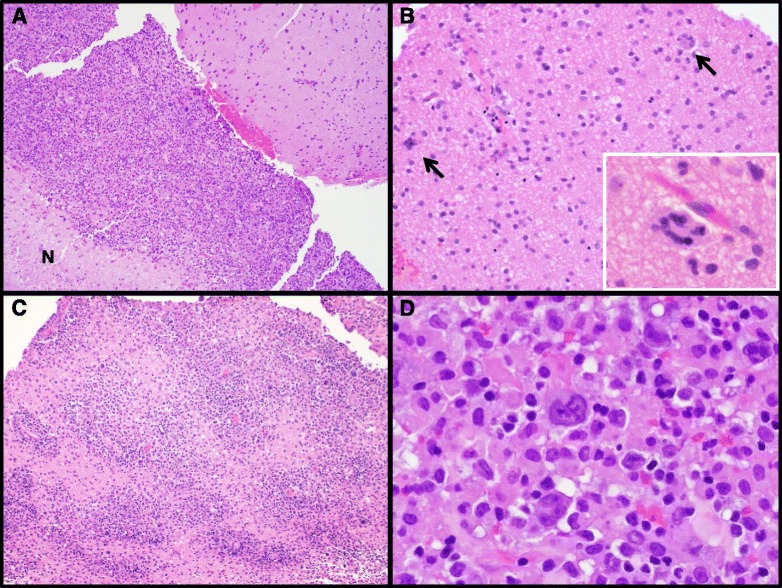
H&E Stained Sections. Photomicrographs of H&E stained sections showing fragments of brain tissue with distinct areas of gliosis, hypercellularity, and necrosis (N) at 40X magnification (Part **a**). The gliotic brain exhibited perivascular multinucleated giant cells (arrows) (**b**, 200X, and inset, 400X), while the hypercellular areas were comprised of polymorphous atypical cells with occasional angiocentric architecture (**c**, 200X) and large, Reed-Sternberg-like cells (**d**, 400X)

Permanent microscopic sections were then reviewed (Fig. 2), and showed fragments of brain parenchyma with a diffuse mild astrogliosis and scattered multinucleated giant cells, mostly in a perivascular distribution, consistent with HIV encephalitis. In addition, there was a well-demarcated region of hypercellularity composed of polymorphic cells, corresponding to the area noted at frozen section diagnosis. This area contained small, mature lymphocytes, medium-sized cells with irregular nuclei and abundant cytoplasm, and very large cells with hyperchromatic, bizarre nuclei, one or more eosinophilic nucleoli, and abundant eosinophilic cytoplasm, reminiscent of Reed-Sternberg or Hodgkin cells. This polymorphic population of cells was arranged in a diffuse sheet in one area, but was also perivascular in other areas, and formed a rim around a small area of necrosis. Again, acute inflammation and microorganisms were absent. Differential diagnostic considerations included multifocal glioblastoma, lymphoma, and HIV or other viral-related reactive changes.

**Fig. 3:**
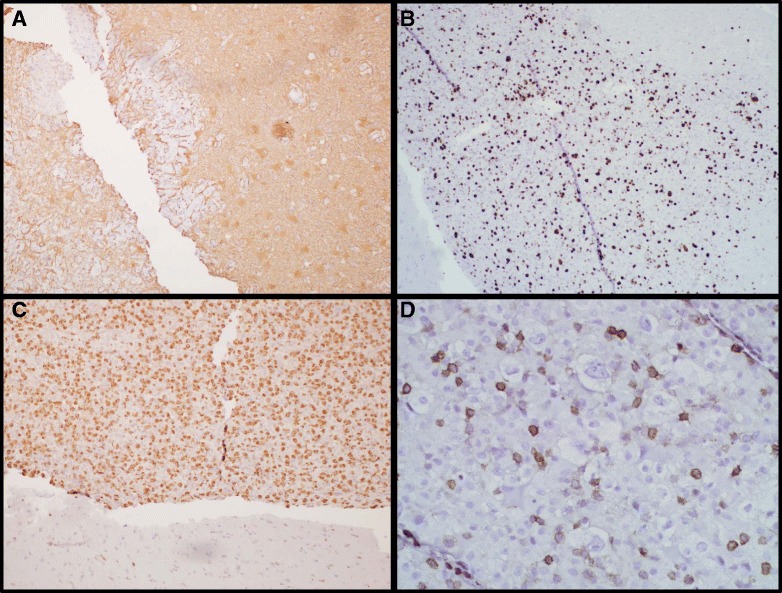
GFAP, MIB-1, CD68, and CD45 Immunohistochemistry. Photomicrographs of immunohistochemical stains show a reactive pattern for glial acidic fibrillary protein (GFAP) in the background brain, but no significant staining in the hypercellular lesion (**a**,40X). The hypercellular region exhibits significantly increased MIB-1 staining (**b**,100X), strong CD68 positivity (**c**,100X), and only rare, small, CD45-positive cells (D,400X) compared to the background brain

Immunohistochemistry was initially performed for glial fibrillary acidic protein (GFAP), MIB-1, CD45, CD68, CMV, and toxoplasmosis antigen (Fig. 3). GFAP was positive in reactive astrocytes within the background brain, but negative in the atypical cells. CD45 highlighted only scattered small cells in the hypercellular areas. CD68 was diffusely positive in many of the medium-to-large epithelioid cells in the hypercellular areas, as well as in the giant cells in the background brain parenchyma. MIB-1 was positive in approximately 50 % of the atypical cells, while stains for CMV and toxoplasmosis were negative. Gram, Grocott’s Methenamine Silver (GMS), acid fast bacilli (AFB), and Fite special stains were also negative. These findings, in particular the CD68 positivity and pathognomonic multinucleated giant cells in the background, pointed away from a neoplastic glial or lymphomatous process and toward a reactive histiocytic phenomenon associated with HIV infection.

**Fig. 4:**
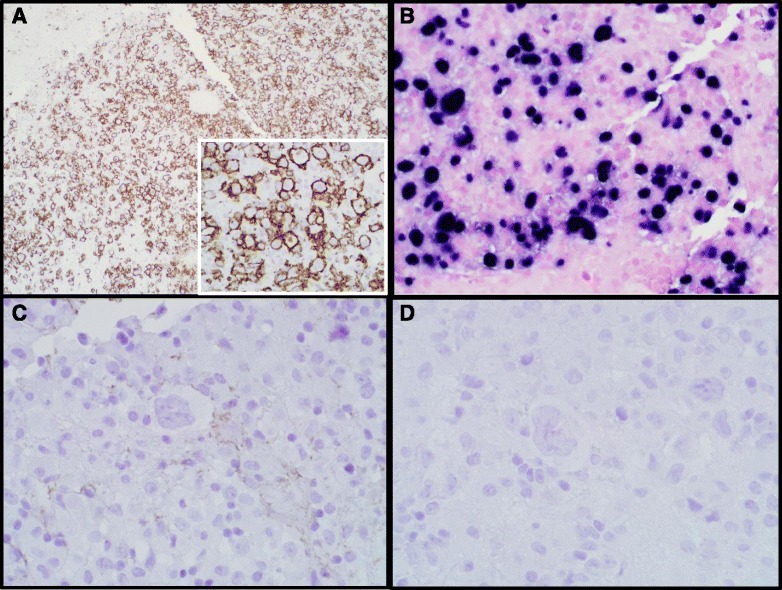
CD20, CD15, and CD30 Immunohistochemistry and EBV *In Situ Hybridization.* Photomicrographs of the hypercellular lesion show that the large, Reed-Sternberg-like cells and many intermediate-sized cells were strongly positive for CD20 by immunohistochemistry (**a**,100X, and inset,400X), and EBV via *ISH* (**b**, 400X); the large cells were negative for CD15 (**c**, 400X) and CD30 (**d**, 400X) by immunohistochemistry

**Fig. 5:**
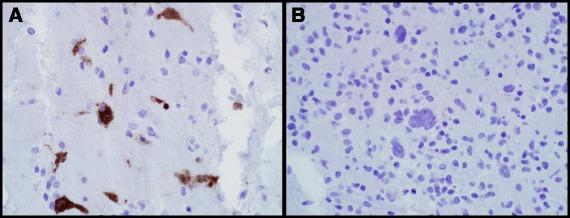
P24 Immunohistochemistry. Photomicrographs of the background brain show positive p24 immunohistochemical staining within the multinucleated giant cells of HIV encephalitis (**a**, 400X), while the neoplastic cells are negative (**b**, 400X)

However, in an effort to rule out rare CD45-negative lymphomas, and due to the striking Reed-Sternberg-like morphology, additional stains for CD15, CD30, CD20, CD79a, and PAX5 were performed. EBV testing via *in situ hybridization* (ISH), and a p24 immunostain were also obtained. The large, Reed-Sternberg-like atypical cells, along with most of the medium-to-large cells, were strongly positive for CD20, CD79a, and EBV, and negative for CD15 and CD30 (Fig. 4). The majority of these cells were also positive for MUM1, focally and weakly positive for PAX5 and BCL6, and negative for CD10. B-cell clonality testing (Polymerase Chain Reaction-based IGH + IGK B-Cell Clonality Assay™, Invivoscribe Technologies Inc, San Diego, CA, USA) yielded a clonal result. P24 was positive in scattered clusters of multinucleated giant cells in the background brain, but negative in the CD20-positive cell population (Fig. 5). In light of this staining pattern, the diagnosis of HIV-associated lymphoproliferative disorder was rendered alongside a diagnosis of HIV encephalitis. The neoplasm was subclassified as a Diffuse Large B-Cell Lymphoma (DLBCL) of post-germinal center origin (MUM1-positive).

Several days after the biopsy, the patient was started on combination anti-retroviral therapy (cART), but developed additional complications. After discussion with the family, the patient was transitioned to palliative care and died 3 weeks following the biopsy. An autopsy was not performed.

## Discussion

This case presented several unique and interesting diagnostic challenges, starting with the initial laboratory testing and flow cytometry results. The clinical presentation and brain imaging allowed for a focused differential diagnosis including common HIV-associated infectious and neoplastic disorders. However, one of the first barriers to diagnosis was the lack of EBV-DNA detected in the patient’s CSF. The sensitivity (80-100 %) and specificity (93-100 %) of EBV-DNA testing by PCR in the CSF have traditionally been considered quite high for PCNSL, making it a very useful diagnostic marker [6]. Though a recent study showed a more realistic sensitivity of 70 % and a specificity of 85 % [4], the negative result in this case is still a less common result and one that argued against a diagnosis of PCNSL. Similarly, flow cytometric analysis, performed on a fragment of the brain biopsy, was uninformative, most likely due to sampling error or neoplastic cell size.

One of the most challenging aspects of this case was determining whether the pathologic process was truly neoplastic or within the spectrum of reactive HIV- or EBV-related changes. In our case, the majority of the lesional cells were initially found to be strongly positive for CD68 and negative for CD45. This immunophenotype is most consistent with a histiocytic infiltrate, and in the setting of pathognomonic HIV encephalitis with CD68-positive multinucleated histiocytes, would favor a reactive process due to HIV infection. The most significant diagnostic pitfall would have been to then exclude a lymphoproliferative disorder on the basis of a CD45-negativity.

Though commonly employed as a first-line hematolymphoid lineage-specific immunostain, the subset of lymphoproliferative disorders lacking CD45 expression is well-established. Immunostaining for CD45 is typically negative in Reed-Sternberg cells in classic Hodgkin lymphoma, the majority of plasmacytic neoplasms, approximately 10 % of precursor B-cell neoplasms, some anaplastic large cell lymphomas, and rarely other non-Hodgkin lymphomas including DLBCL [8, 9]. The key to the correct diagnosis in this case was the morphologic recognition of large, atypical Reed-Sternberg-like cells. Though difficult to miss, these cells also possessed features of viral cytopathic effect, again leading to consideration of a purely reactive phenomenon. However, their overall cytologic atypia led to a thorough immunohistochemical work-up, identifying EBV-infected CD20-positive B-cells, consistent with an HIV-associated B-cell lymphoproliferative disorder.

Subclassification of this neoplasm presented a final, interesting challenge in this case. PCNSLs are histologically defined using the World Health Organization (WHO) Classification of Tumours of Haematopoietic and Lymphoid Tissue [5], which separates AIDS-related lymphomas into three general categories: 1) lymphomas also occurring in immunocompetent patients, 2) lymphomas occurring more specifically in HIV-positive patients, and 3) lymphomas also occurring in other immunodeficiency states. The most common subtype of AIDS-related PCNSL is reportedly diffuse large B-cell lymphoma with immunoblastic or plasmacytic differentiation, but Burkitt and Burkitt-like subtypes as well as polymorphous unclassifiable neoplasms have also been reported [6, 7]. Interestingly, another pathologic entity termed the Atypical Lymphoid Proliferation, not currently described by the WHO, has been used to describe polyclonal proliferations of pleomorphic, CD20, EBV-positive lymphocytes in cardiac myxomas [10]. These cases share striking morphologic similarities with our case as well as EBV positivity, and may represent a pre-clonal entity sharing a similar pathogenetic mechanism tied to EBV and a chronic inflammatory state.

Our case exhibited several features of Hodgkin lymphoma including striking Reed-Sternberg-like morphology, a CD45-negative immunophenotype, EBV positivity, and a background of small, mature lymphocytes and numerous histiocytes. Though being a well-documented subtype of AIDS-related systemic lymphoma, Hodgkin lymphoma has not been reported as a subtype of AIDS-related PCNSL. Our case also exhibited strong CD20 expression in both the large and intermediate-sized cells, and lacked CD15 and CD30 expression characteristic of Hodgkin lymphoma. While morphologically consistent with Hodgkin lymphoma, the immunophenotype in this case led to its final classification as diffuse large B-cell lymphoma.

Regardless of the immunophenotypic classification, the prognosis of patients with AIDS-related PCNSL is unfortunately dismal, with a median survival of 1 to 2.5 months with supportive care alone [6]. Whole-brain radiation is the most common treatment employed, and has been reported to increase median survival from 2 to 5.5 months [11]. Patients have also been treated with antiretroviral therapy (HAART) without significantly prolonged survival [6]. Chemotherapy is typically contraindicated due to the severe risk of infection in these immunocompromised patients. Our patient, unfortunately, also experienced a characteristically poor outcome.

## Conclusions

We have reported a challenging case of an HIV-associated lymphoproliferative disorder with unusual morphologic features reminiscent of Hodgkin Lymphoma. This case highlights several common and important diagnostic pitfalls that can occur in the work-up of patients with HIV and brain abnormalities on imaging. Early diagnostic pitfalls included negative CSF EBV testing, likely due to the insensitivity of this test, and negative flow cytometric analysis, which can easily result from sampling error. Upon biopsy, the pathologic diagnosis was complicated by a CD45-negative immunophenotype and Hodgkin-like morphology. In fact, this is the first known report of this unique morphology in the setting of an HIV-related primary CNS lymphoproliferative disorder, and is of unknown clinical significance. We hope that improved understanding of the pathology of these neoplasms may lead to timely, appropriate, and perhaps earlier, diagnosis and improved treatment options.

## Consent

Written informed consent was obtained from the patient’s next of kin and power of attorney for publication of this Case Report and any accompanying images. A copy of the written consent is available for review by the Editor-in-Chief of this journal.

## Abbreviations

PCNSL: Primary CNS Lymphoma
HIV: Human Immunodeficiency Virus
AIDS: Acquired Immunodeficiency Syndrome
DLBCL: Diffuse Large B-Cell Lymphoma
WHO: World Health Organization
EBV: Ebstein-Barr Virus
CMV: Cytomegalovirus
HSV: Herpes Simplex Virus
VZV: Varicella Zoster Virus
HAART: Highly Active Anti-Retroviral Therapy
cART: Combination Anti-Retroviral Therapy
GMS: Grocott’s Methenamine Silver
AFB: Acid Fast Bacilli
CSF: Cerebrospinal Fluid
